# Latent class analysis to evaluate performance of point-of-care CCA for low-intensity *Schistosoma mansoni* infections in Burundi

**DOI:** 10.1186/s13071-018-2700-4

**Published:** 2018-02-23

**Authors:** Michelle N. Clements, Paul L. A. M. Corstjens, Sue Binder, Carl H. Campbell, Claudia J. de Dood, Alan Fenwick, Wendy Harrison, Donatien Kayugi, Charles H. King, Dieuwke Kornelis, Onesime Ndayishimiye, Giuseppina Ortu, Mariama Sani Lamine, Antonio Zivieri, Daniel G. Colley, Govert J. van Dam

**Affiliations:** 1 Schistosomiasis Control Initiative (SCI), Department of Infectious Disease Epidemiology, Imperial College London, London, UK; 2 Department of Molecular Cell Biology, Leiden University Medical Center, Leiden, the Netherlands; 3 Center for Tropical and Emerging Global Diseases, University of Georgia, Athens, GA USA; 4 Programme National Intégré de lutte contre les Maladies Tropicales Négligées et la Cécité (PNIMTNC), Ministère de la Santé Publique et de la Lutte contre le SIDA, Bujumbura, Burundi; 5 Center for Global Health and Diseases, Case Western Reserve University School of Medicine, Cleveland, OH USA

**Keywords:** Schistosomiasis, Diagnostics, Latent class analysis, CCA, CAA, Kato-Katz

## Abstract

**Background:**

Kato-Katz examination of stool smears is the field-standard method for detecting *Schistosoma mansoni* infection. However, Kato-Katz misses many active infections, especially of light intensity. Point-of-care circulating cathodic antigen (CCA) is an alternative field diagnostic that is more sensitive than Kato-Katz when intensity is low, but interpretation of CCA-trace results is unclear. To evaluate trace results, we tested urine and stool specimens from 398 pupils from eight schools in Burundi using four approaches: two in Burundi and two in a laboratory in Leiden, the Netherlands. In Burundi, we used Kato-Katz and point-of-care CCA (CCAB). In Leiden, we repeated the CCA (CCAL) and also used Up-Converting Phosphor Circulating Anodic Antigen (CAA).

**Methods:**

We applied Bayesian latent class analyses (LCA), first considering CCA traces as negative and then as positive. We used the LCA output to estimate validity of the prevalence estimates of each test in comparison to the population-level infection prevalence and estimated the proportion of trace results that were likely true positives.

**Results:**

Kato-Katz yielded the lowest prevalence (6.8%), and CCAB with trace considered positive yielded the highest (53.5%). There were many more trace results recorded by CCA in Burundi (32.4%) than in Leiden (2.3%). Estimated prevalence with CAA was 46.5%. LCA indicated that Kato-Katz had the lowest sensitivity: 15.9% [Bayesian Credible Interval (BCI): 9.2–23.5%] with CCA-trace considered negative and 15.0% with trace as positive (BCI: 9.6–21.4%), implying that Kato-Katz missed approximately 85% of infections. CCAB underestimated disease prevalence when trace was considered negative and overestimated disease prevalence when trace was considered positive, by approximately 12 percentage points each way, and CAA overestimated prevalence in both models. Our results suggest that approximately 52.2% (BCI: 37.8–5.8%) of the CCAB trace readings were true infections.

**Conclusions:**

Whether measured in the laboratory or the field, CCA outperformed Kato-Katz at the low infection intensities in Burundi. CCA with trace as negative likely missed many infections, whereas CCA with trace as positive overestimated prevalence. In the absence of a field-friendly gold standard diagnostic, the use of a variety of diagnostics with differing properties will become increasingly important as programs move towards elimination of schistosomiasis. It is clear that CCA is a valuable tool for the detection and mapping of *S. mansoni* infection in the field and CAA may be a valuable field tool in the future.

**Electronic supplementary material:**

The online version of this article (10.1186/s13071-018-2700-4) contains supplementary material, which is available to authorized users.

## Background

The standard field method of detecting *Schistosoma mansoni* is through Kato-Katz (KK) testing, in which patient stool samples are examined by microscopy for parasite eggs [1, 2]. However, this method is well known to miss some infections, especially those of light intensity [3]. Consequently, KK-based surveys can significantly underestimate local prevalence of infection, especially when the average infection intensity is low within the communities tested [4], and when Kato-Katz sampling does not take place over a number of days.

The point-of-care circulating cathodic antigen (CCA) test is a field diagnostic test for *S. mansoni* that detects circulating antigen from intravascular *Schistosoma* adult worms, which is excreted in the urine of infected people. CCA testing has recently been accepted by the World Health Organisation for use in programmatic ‘mapping’ in areas where *S. mansoni* is the only endemic *Schistosoma* species [5]. CCA testing has been shown to detect a higher proportion of *S. mansoni* infections than KK testing [6], especially where infection is present at low intensities [4, 7], and the CCA test exhibits less day-to-day variation than KK [8]. However, whether faint ‘trace’ results should be considered as negative or positive has been questioned [9–11].

Circulating Anodic Antigen (CAA) is another *Schistosoma* antigen. The laboratory-based Up-Converting Phosphor-Lateral Flow CAA assay can be used on serum (or plasma) or urine [12, 13]. Testing for CAA is more sensitive at lower intensities of infection than either KK or CCA [14], and CAA performed on a 2 ml sample of urine (UCAA2000) may potentially detect a single worm pair [13, 15]. Testing for the CAA antigen is also highly specific [16]. Data from low-endemic settings in Zanzibar (*Schistosoma haematobium* [17]) and China (*Schistosoma japonicum* [18]) indicate that the percentage of *Schistosoma* infected individuals as determined by UCAA2000 testing may be an accurate measure of prevalence of ongoing active infections.

Although the CAA test appears a potential candidate, there yet is no generally accepted ‘gold standard’ test to definitively categorise individuals as truly infected (positive) or truly uninfected (negative). Latent class analysis (LCA) is a statistical technique commonly used to compare the performance of diagnostic tests in the absence of a gold standard test. It has been used in a number of different fields, including tuberculosis [19], malaria [20], veterinary biology [21] and schistosomiasis [17]. LCA uses all available information to estimate the proportion of true positives testing positive for each test (i.e. the sensitivity of each test), the proportion of true negatives testing negative for each test (i.e. the specificity of each test), and the proportion of individuals truly positive in the population (i.e. the infection prevalence within the population), as reviewed in [22, 23]. Applying LCA within a Bayesian framework enables straightforward assessment of the prevalence estimates from each test by comparing the distributions of estimated test and estimated infection prevalence. Here we use LCA to compare different diagnostic tests (Table 1) to assess the performance of the CCA test in low intensity settings, with particular focus on how to interpret trace results.

**Table 1:** Description of the diagnostics assessed

Short code	Diagnostic	Where assessed	Sample used	Metric	Definition of positive
KK	Kato-Katz	Schools in Burundi	2 × 1/24th g slides from a single stool sample	Number of *S. mansoni* eggs detectable by visual microscopy	Any *S. mansoni* egg found in either slide
CCABtn	Point-of-care circulating cathodic antigen	Schools in Burundi (CCAB)	Urine sample	Intensity of band against reference	1/2/3
CCABtp	Point-of-care circulating cathodic antigen	Schools in Burundi (CCAB)	Urine sample	Intensity of band against reference	trace/1/2/3
CCALtn	Point-of-care circulating cathodic antigen	Laboratory in Leiden (CCAL)	Urine sample	Intensity of band against reference	1/2/3
CCALtp	Point-of-care circulating cathodic antigen	Laboratory in Leiden (CCAL)	Urine sample	Intensity of band against reference	trace/1/2/3
CAA	Up-converting phosphor-lateral flow circulating anodic antigen	Laboratory in Leiden	Urine sample	Laboratory test reader (strip scanner)	≥ 1 pg CAA/ml urine

Burundi has had a national control programme for *S. mansoni* since 2008, in which school-age children are treated with praziquantel during the nation’s annual maternal health weeks [24, 25]. In 2014, Burundi undertook a mapping exercise, visiting 347 schools throughout the country, testing 50 children within each school using the CCA test [26], with the expectation that repeated rounds of treatment would have lowered *S. mansoni* prevalence. One of the aims of the remapping study was to assess the suitability of Burundi for an elimination project by the Schistosomiasis Consortium for Operational Research and Evaluation (SCORE; https://score.uga.edu/). As shown in Ortu, Ndayishimiye et al. [26], prevalence of *S. mansoni* by CCA performed in Burundi (CCAB) was 13.5% if ‘trace’ readings were considered negative (CCABtn), rising to 42.8% when ‘trace’ readings were considered positive (CCABtp). Concurrently, KK tests were performed in 170 of these 347 schools to assess soil transmitted helminths and *S. mansoni* infections. Within these 170 schools, prevalence of *S. mansoni* by KK was 1.5% and prevalence by CCAB was 10.9% when ‘trace’ was considered negative, rising to 41.3% when ‘trace’ was considered positive. Of the children who were tested by KK, only 1 one out of 8482 (0.01%) was heavily infected according to WHO guidelines (i.e. having ≥ 400 eggs per gram (epg) of stool [1]), and 18 children (0.21%) were assessed as moderately infected (100–399 epg). The remaining KK egg-positive children had light intensity infections (1–99 epg). No children tested positive by KK in 84% of schools tested; the corresponding figures for CCA were 18% when trace was considered negative and just 2.4% when trace was considered positive.

Subsequently, urine samples from eight purposively selected schools were sent to Leiden University Medical Center in the Netherlands, where they were tested again using CCA cassettes from the same batch as was used in Burundi, and by the CAA assay. The present LCA analysis uses the test results from these eight more intensively studied schools with the following aims: (i) To assess the sensitivity and specificity of four test results: KK egg detection performed in Burundi, CAA performed in Leiden, and CCA performed independently in both Burundi (CCAB) and Leiden (CCAL). For the latter CCA tests, we also compared outcomes when a ‘trace’ result was considered either negative (CCAtn) or positive (CCAtp). (ii) To evaluate which test gave prevalence estimates closest to the LCA-estimated infection prevalence. (iii) To estimate the proportion of CCA trace results that were true positives. (iv) To estimate test properties and infection prevalence if the CAA positive results were taken to be truly positive (i.e. assuming CAA was 100% specific).

## Methods

### Data collection

The process used to select children for testing in the remapping survey has been explained in detail in Ortu et al. [26], and will only be briefly discussed here. We divided Burundi into five ‘ecological zones’ and determined the number of schools to be tested in each zone based on historic data. Within each zone, schools to include in the study were selected randomly for testing with CCA only, KK only, or both CCA and KK. In each school, 50 children between 13 and 14 years of age present at school on the testing day were randomly selected for testing, with no reference to possible infection status; if 50 children aged 13–14 years were not available, ‘top-up’ from between ages 12–16 was permitted, with selection of the remaining children being random with no reference to age or possible infection status. KK microscopy was done on a single stool sample, for which two separate slides were prepared and examined and the number of eggs on each slide recorded. CCA testing was performed on a single urine sample taken on the same day as the stool collection and scored on a five-point scale: negative, trace, positive 1, positive 2, and positive 3. Residual urine from sampled children in all schools was stored at -20 °C for potential analysis in Leiden. CCA data was available for 347 schools, with 170 of these schools also having KK data.

Following review of the remapping study results (see introduction), eight schools were purposively selected from among the 170 schools with both KK and CCA results to have their stored urine samples sent to Leiden for confirmatory CCA and CAA testing. The sample size of eight schools (approximately 400 pupils) was driven by cost considerations. Only schools that were tested using both CCA and KK (170 schools) were eligible for selection to be sent to Leiden so that each child could be assessed with all four tests. Random sampling of schools was not felt to be appropriate as only 16% of the 170 schools [26] had at least one child positive by KK, and consequently there was a risk that the KK data would not be informative in subsequent analyses. However, simply choosing the eight schools with the highest prevalence by KK (> 10% prevalence) would clearly also not be appropriate as the results would not be representative of the population. Additionally, much of the focus of discussion around the field results was related to the large number of trace CCA results obtained and there was a desire to investigate these further. Given the above considerations, schools were then selected following review of the data that had sufficient numbers of urine samples from children positive by KK, as well as from children who were negative by KK but positive by CCAB. Because assessment of trace results was a priority, efforts were made to include an adequate number of children who had trace-positive CCA readings, where ‘sufficient’ and ‘adequate’ were determined by consensus by the project team. The selected urine samples were anonymised, blinded, and sent to Leiden by commercial shipper, maintained at 4 °C throughout transit.

Upon arrival in Leiden, the specimens were stored at -20 °C until testing. The CCA and CAA assays were run independently by technicians blinded to the results obtained in Burundi, and using the same batch of CCA tests as used in Burundi. The CCA test was performed as described by the manufacturer with one drop (40 μl) of urine, and negative/trace/positive scores were based upon comparison with a reference cut-off sample. The CAA assays were performed as described elsewhere using 2 ml urine [13, 15, 27].

### Determining sensitivity and specificity of each test

Data were analysed using LCA, with one model in which a CCA trace-positive was considered as negative for *S. mansoni* infection, and one in which it was considered positive. The details of LCA are explained in many recent publications (e.g. [22, 23]) and we will only describe it briefly here. The first step in an LCA analysis is to calculate the number of people with each test combination (positive for all four tests, positive by KK but negative by all other tests, etc.). Each test combination is then related to the sensitivity and specificity of each test, and the infection prevalence in the population (the ‘latent class’ of interest) using likelihood functions.

These likelihood functions relate the diagnostic test parameters being estimated to the observed test results. Given each individual’s assumed latent infection status, each test correctly or incorrectly determines them to be positive or negative. For truly positive individuals, the probability of each test correctly determining them to be positive is the sensitivity of the test, whereas for truly negative individuals, the probability of each test correctly determining them to be negative is the specificity of the test, with the proportion of truly positive and truly negative being determined by the infection prevalence in the population. The likelihood equation for each test result combination has two components: the probability of each test combination for truly positive individuals (a function of the test sensitivities and the prevalence) and the probability of each test combination for truly negative individuals (a function of test specificities and 1 minus the prevalence, representing the proportion of uninfected people in the population). The likelihood functions are then related to the observed test combinations through a multinomial distribution. The likelihood equations can be extended to incorporate covariance terms between the sensitivities and specificities of different tests, reflecting conditional non-independence of tests, for instance when both tests perform better at detecting high intensity infections than low intensity infections [28]. Additionally, LCA can be further extended to incorporate different populations, which also allows for greater degrees of freedom in the analysis [29].

We ran the analyses in a Bayesian Framework using OpenBugs [30] through the R2OpenBugs package [31] in R [32]. We used Beta priors on the sensitivity and specificity of each test to restrict possible values to between 0 and 1 using the BetaBuster application [33], with the strongest prior being placed on KK test specificity (Table 2). The independent prevalence estimates in each school each had a prior of a Beta distribution with α = β = 1, which is equivalent to the uniform distribution on the interval [0, 1]. We took each school to be a different population and linked the likelihood functions to the test combinations using the multinomial distribution. See Additional file 1: Code S1 for the code used in the analysis. We ran the models using three chains, each with 1000 iterations, a thin of 25, and a burn-in of 100, giving an output of 2700 iterations. (The OpenBugs iteration parameter specifies the number of iterations after thinning but before the burn-in. Consequently, each chain returned 1000–100 = 900 iterations.) Convergence of the models was assessed using Gelman diagnostics [34], and the appropriateness of the model fit was assessed by comparing observed test prevalence to the denominator of the positive predictive value (PPV): values of, for example, KK prevalence far above the observed 6.8% indicated that the model had converged to a local maximum rather than a global maximum. Convergence to a local maximum happened more often when multiple covariances were fitted in the model. This was not an issue in our final model.

**Table 2:** Details of informative priors used in Bayesian latent class analysis for test parameters. Alpha and Beta denote the parameters of the beta distribution and the mean, standard deviation (SD), 95% greater than and mode indicate the properties of the distribution with the given parameters. The sum of Alpha and Beta is often known as the ‘sample size equivalent’ and the effect of the prior can be thought of as adding alpha + beta samples to the analysis, with alpha samples being positive

Parameter	Test	Alpha	Beta	Mean (%)	SD (%)	95% greater than (%)	Mode (%)	Sample size equivalent
Sensitivity	KK	1.43	1.29	53	26	10	60	2.72
	CCAB	3.05	1.51	53	26	30	80	4.56
	CCAL	3.05	1.51	53	26	30	80	4.56
	CAA	3.05	1.51	53	26	30	80	4.56
Specificity	KK	21.2	2.06	91	6	80	95	23.26
	CCAB	5.38	1.49	53	26	50	90	6.87
	CCAL	5.38	1.49	53	26	50	90	6.87
	CAA	5.38	1.49	53	26	50	90	6.87

Covariances between each pair of tests were included in the model: the covariance was added to the sensitivity or specificity term when the test results were both positive or both negative and subtracted when the test results were different. We attempted to fit covariance terms between all tests in the model but we ran into convergence challenges (see above), presumably due to the low number of samples in the analysis. Instead, we removed some covariances by setting the parameter values to zero in the model. We fitted covariances of each separate pair of tests in turn and assessed the Bayesian Deviance Information Criterion (DIC) of the resultant models (Additional file 1: Table S1). We always fitted covariances between both the sensitivity and specificity of the same tests, and uniform priors were specified for the covariances, with each covariance being bounded between zero and an upper limit such that the relevant term containing the covariance could not go above one. Following the single covariance terms model, we then tested whether the two models with the lowest DIC gave a lower value when both covariance terms were fitted in the model. We also attempted to fit trace negative and trace positive outcomes in a single model (through specifying trace positive to be the same or more sensitive and the same or less specific than trace negative) but this led to some parameter values becoming fixed at the boundary values of zero or one resulting in a zero estimate of error. We tested the sensitivity of the final model to the choice of priors by replacing, in turn, the prior for the sensitivity or specificity by the uniform distribution (Beta(1, 1)), and also by replacing priors for all sensitivities and specificities by Beta(1, 1) in the same model.

### Comparing estimated test and estimated infection prevalence

One of the advantages of analysis within a Bayesian framework is that the posterior distribution can easily be used to calculate the distribution of ancillary variables from the different iterations. We calculated the estimated infection prevalence in the population using a weighted average (weighted for the number of pupils within each school) of the estimated infection prevalence from each school.

We then compared the estimated infection prevalence with each test prevalence by simulating an error distribution around each observed test prevalence. We obtained a distribution of the number of children testing positive for each school and test by sampling 2700 times (once for each iteration from the posterior distribution) from a binomial distribution with parameters n equal to the number of children tested in the focal school (49 or 50) and p equal to the proportion of children testing positive in the focal school using the focal test. An overall estimate of prevalence by each test was then obtained by summing the simulated number of positive children across all schools and dividing by the total number of children tested. Estimated infection prevalence and estimated test prevalence were then compared using subtraction on the 2700 iterations of each parameter.

### Estimating the proportion of trace results that were truly positive

The PPV of a test estimates the probability that an individual testing positive is truly positive. The PPV for test i can be calculated using the equation:

$$ {PPV}_i=\frac{prev\ast {Se}_i}{prev\ast {Se}_i+\left(1- prev\right)\ast \left(1-{Sp}_i\right)} $$

As much of the discussion around trace CCA results have focused on the proportion of trace results that are truly positive, we then used the output from both models together to estimate the PPV of the trace results alone using the equation:

$$ {PPV}_{trace}=\frac{prev_{trace\_ pos}\ast {PPV}_{trace\_ pos}-{prev}_{trace\_ neg}\ast {PPV}_{trace\_ neg}}{prev_{trace\_ pos}-{prev}_{trace\_ neg}} $$

where *PPV*_*trace*_*neg*_ and *PPV*_*trace*_*pos*_ denotes the PPV when traces were considered negative and positive respectively, calculated using the equation above, and *prev*_*trace*_*neg*_ and *prev*_*trace*_*pos*_ denotes the prevalence estimates obtained from the LCA when traces were considered negative and positive, respectively.

### Estimating test properties when specificity of CAA fixed at 100%

We re-ran all of the analyses above with the specificity of CAA fixed to 100% to determine test properties if it was assumed that all of the CAA positive results were true positives.

## Results

### Prevalence estimates and test combinations

The dataset consisted of data from 398 pupils from eight different schools, with each child having four separate test results, and six separate test readings after inclusion of the two interpretations of each CCA result.

There was an almost eight-fold difference between the highest and lowest prevalence estimates by the studied tests for *S. mansoni*, ranging from 6.8% by KK to 53.5% by CCABtp (Table 3, Additional file 1: Table S2 for prevalence figures in each school). The most striking difference between CCA results in Burundi and Leiden were the number of trace results recorded: almost one-third (32.4%) of children tested in Burundi were recorded as trace by CCA compared to only 2.3% in Leiden. Of the nine pupils assessed as trace by CCAL, eight were also trace in Burundi, and one was assessed as positive 1 (Additional file 1: Table S3). Of the 129 pupils assessed as trace by CCAB, 88 were negative by CCAL, eight were trace, 31 were positive 1, two were positive 2 and none were positive 3. When children assessed as trace in either Burundi or Leiden were removed from the results, 84% of the remaining test results were the identical by both CCAB and CCAL, and 97% were within one point on the marking scale.

**Table 3:** Summary statistics and prevalence by different diagnostic tests in eight schools in Burundi. Samples from each child were tested using four different diagnostics: Kato-Katz performed in Burundi, CCA performed in Burundi, CCA performed in Leiden and CAA performed in Leiden. Additionally, CCA results were considered both with trace results as negative and with trace results positive

Test	Prevalence (%) (by school range)^a^
Kato-Katz in Burundi (KK)	6.8 (0–20)
CCA in Burundi: trace-negative (CCABtn)	21.1 (0.0–44.0)
CCA in Burundi: trace-positive (CCABtp)	53.5 (12.0–90.0)
CCA in Leiden: trace-negative (CCALtn)	28.4 (0.0–60.0)
CCA in Leiden: trace-positive (CCALtp)	30.7 (0.0–64.0)
CAA in Leiden (CAA)	46.5 (6.0–78.0)

^a^Data from 398 pupils from 8 schools. Between 48% and 59% of children tested within each school were female, with the overall average being 51%. The mean age of children was 13.1 years with an associated standard deviation of 0.67 years

**Fig. 1:**
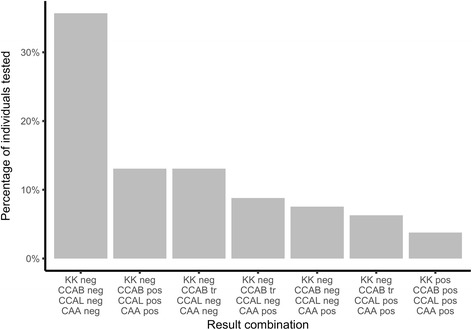
Combinations of *Schistosoma mansoni* diagnostic test results from 398 pupils in Burundi. The graph shows the percentage of pupils with each test result combination for those test combinations with at least 10 pupils. The most frequent test combination was negative by all four tests. *Abbreviations*: Neg, negative; tr, trace; pos, positive. All other abbreviations as defined in Table 1

LCA uses the test result combinations for each studied individual to estimate the diagnostic performance of each included test. The most frequent test combination was negative by all four tests (35.7%; Fig. 1). In contrast, positive by all four tests was only the seventh most common test combination (3.8%). The test combinations between 1st and 7th most common were all negative by Kato-Katz but trace or positive by one or more antigen tests. See Additional file 1: Table S4 for all test combinations, overall and split by school. Of the 27 children who were positive by KK, 16 (59%) were also positive by CCAB when trace was considered negative, rising to 22 (81%) when trace was considered positive; CCAL was positive in 21 of the 27 (78%); since no KK-positive child had a CCAL trace result, this was true whether trace was considered negative or positive. CAA was positive for 24 of the 27 KK-positive children (89%). A total of 185 children were assessed to be positive by CAA. Of these 185 children, fewer were positive by CCAB (41%) than CCAL (56%) when trace was negative, but more were positive by CCAB (79%) than CCAL (59%) when trace was positive. Additionally, of the 213 children negative by CAA, fewer were positive by CCAB than CCAL both when trace was negative (proportion of CAA negative children positive, CAAB: 4%, CAAL: 35%) and when trace was positive (proportion of CAA negative children positive, CAAB: 31%, CAAL: 36%).

### Sensitivity and specificity estimates

**Fig. 2:**
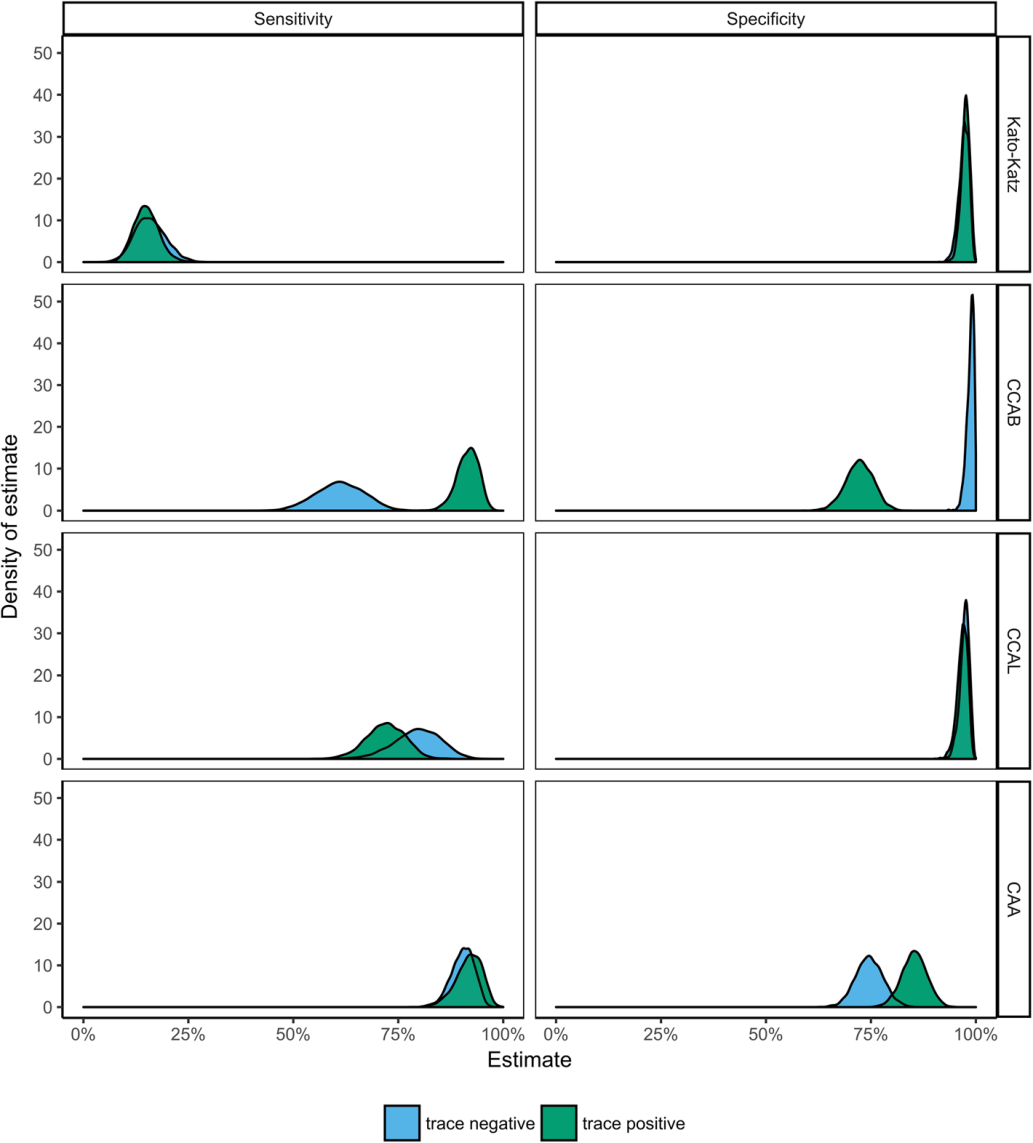
Distribution of sensitivity (left) and specificity (right) of the four diagnostic tests assessed: Kato-Katz, CCA performed in Burundi, CCA performed in Leiden and CAA. Analysis was performed separately for CCA trace as negative (blue) and CCA trace as positive (green). The distribution was obtained by plotting the 2700 iterations outputted from the Bayesian LCA

By far the lowest sensitivity estimate was for KK, at 15.9%, with Bayesian Credible Interval (BCI) 9.2–23.5% when CCA trace readings were considered negative and 15.0% (BCI: 9.6–21.4%) when CCA trace readings were considered positive (Table 4, Fig. 2), implying that KK failed to detect approximately 85% of infections in the study population. The only tests with sensitivity estimates above 90% were CAA (both in the ‘CCA-trace-is-negative’ and the ‘CCA-trace-is-positive’ models) and CCABtp. However, the CAA and CCABtp tests also had the lowest specificity estimates, whereas all other tests had specificity estimates of at least 97%.

**Table 4:** Output from Bayesian LCA to estimate the sensitivity and specificity of diagnostic tests. The mean estimate for each parameter is shown, with 95% Bayesian Credible Intervals (BCI) shown in parentheses. Analysis was performed separately for CCA trace as negative (left) and CCA trace as positive (right). The covariance terms inputted into each model were selected by comparing the DIC’s of models with differing covariance terms added (see Additional file 1: Table S1). The PPV of CCA performed in Burundi was calculated using the equations described in the methods

	CCA trace negative (%) (95% BCI)	CCA trace positive (%) (95%BCI)
Sensitivity		
Kato-Katz in Burundi	15.9 (9.2–23.5)	15.0 (9.6–21.4)
CCA in Burundi	61.1 (49.9–71.9)	91.5 (85.8–96.0)
CCA in Leiden	79.5 (67.7–89.4)	72.0 (62.5–80.5)
CAA in Leiden	90.3 (84.5–95.0)	91.8 (85.0–96.9)
Covariance CCAL & CAA	0.6 (0.0–1.5)	
Covariance CCAB & CCAL		0.3 (0.0–0.7)
Specificity		
Kato-Katz in Burundi	97.1 (94.5–99.1)	97.5 (95.2–99.3)
CCA in Burundi	98.7 (96.6–99.9)	72.3 (65.6–78.7)
CCA in Leiden	97.3 (94.7–99.2)	96.8 (93.9–98.8)
CAA in Leiden	74.6 (68.3–81.2)	85.3 (79.3–91.1)
Covariance CCAL & CAA	0.4 (0.0–1.1)	
Covariance CCAB & CCAL		0.3 (0.0–0.8)
PPV of CCA in Burundi (95% BCI)		
Trace as negative	95.8 (89.4–99.6)	
Trace as positive	69.4 (61.7–7.1)	
Trace results only	52.2 (37.8–5.8)

Considering CCA trace as positive had a large impact on the sensitivity and specificity of CCAB, but less impact for CCAL, presumably due to the lower number of traces in Leiden (Table 4, Fig. 2). The sensitivity and specificity of KK was comparable when trace was considered both as negative and as positive, as was the sensitivity of CAA. However, considering trace as positive led to a substantial increase in the estimate of CAA specificity, from 74.6% (BCI: 68.3–81.2%) when trace was considered negative, to 85.3% (BCI: 79.3–91.1%) when trace was considered positive. See Additional file 1: Table S1 for DICs of the models fitted with different covariance terms and Additional file 1: Table S5 for 95% BCIs of differences between estimates from the trace negative and trace positive models.

### Impact of the choice of priors on the estimates obtained from the LCA

The final model showed very little sensitivity to the choice of priors, even when the priors for all sensitivities and specificities were set to the uniform distribution (Additional file 1: Figure S1). This implies that the analyses were very robust to the choice of priors.

### Comparison of estimated test and estimated infection prevalence

**Fig. 3:**
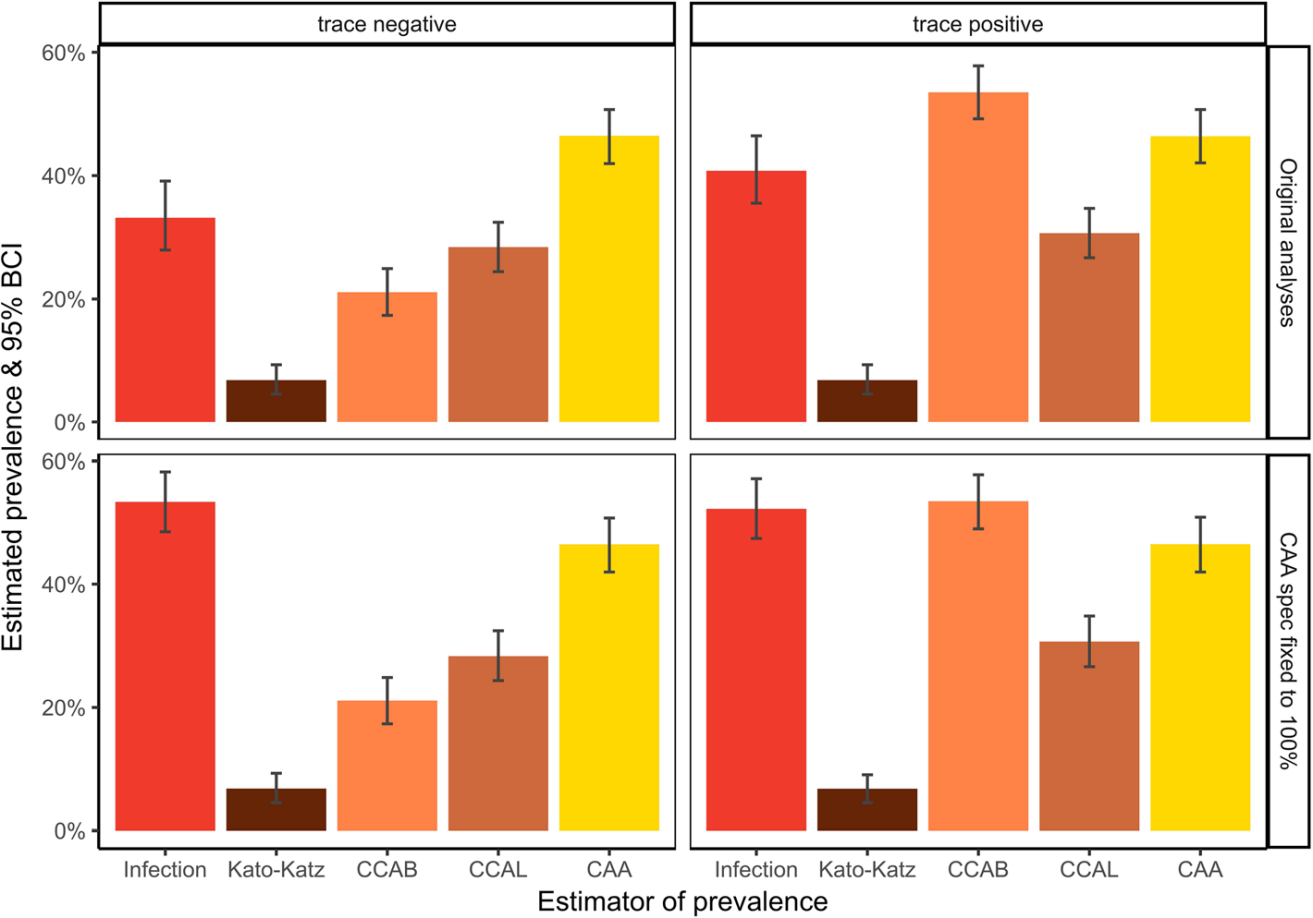
Graphs of infection and test prevalence estimates from 398 pupils in 8 schools. Separate models were run with CCA trace considered as negative (left) and positive (right), both from the original models (top) and models where the specificity of CAA was fixed to 100% (bottom). Infection prevalence estimates were calculated as a weighted average of the prevalence estimate in each school outputted from the Bayesian LCA (weighted for number of pupils sampled in each school). The distribution of estimated test prevalence was obtained by a weighted average of the number of children infected in each school, simulated by drawing from a Binomial distribution with *n* equal to the number of children tested in each school and *p* equal to the proportion of children positive by the focal test

The estimated infection prevalence was 33.1% (BCI: 27.9–39.1%; Table 5; Fig. 3) in the model with trace considered negative, and 40.8% (BCI: 35.5–46,5%) in the model with trace considered positive, although the 95% BCI of difference between the infection prevalence from each model marginally straddled zero (Additional file 1: Table S5). No observed test prevalence was convincingly close to the estimated infection prevalence, but KK was clearly the worst performing test, underestimating prevalence by 26 percentage points when CCA trace was considered negative (BCI: -32.3– -20.7%) and 34 percentage points when CCA trace was considered positive (BCI: -40.0– -28.0%). CCAB underestimated infection prevalence when trace results were considered negative, but overestimated infection prevalence when trace was considered positive, by approximately 12 percentage points each way. CCAL showed a similar pattern, but with much lower amounts of over- and underestimation, and the BCI’s in the model when trace was considered negative suggested that this difference was not significant (overestimation = 4.6%, BCI: -11.9– -1.9%). Both models suggested that CAA overestimated prevalence, although the BCI’s marginally straddled zero in the model when CCA trace was considered positive (overestimation = 5.7%, BCI: -1.4–12.4%).

**Table 5:** The distribution of LCA-estimated disease and test prevalence from 398 pupils in 8 schools. Infection prevalence estimates were calculated as a weighted average of the prevalence estimate in each school outputted from the Bayesian Latent Class Analysis (weighted for number of pupils sampled in each school). The distributions of estimated test prevalences were simulated from the observed prevalence using the binomial distribution. The estimated to infection prevalence gap shows the distribution of estimated infection prevalence minus estimated test prevalence. Separate models were run when CCA trace was considered as negative (left) or positive (right)

	Trace negative (%)				Trace positive (%)			
	Mean	SD	LBCI	UBCI	Mean	SD	LBCI	UBCI
Infection prevalence	33.14	2.88	27.89	39.11	40.78	2.77	35.51	46.48
Estimated prevalence								
Kato-Katz in Burundi	6.79	1.21	4.52	9.30	6.76	1.20	4.52	9.05
CCA in Burundi	21.15	1.95	17.34	24.87	53.58	2.30	49.00	58.04
CCA in Leiden	28.39	2.07	24.37	32.41	30.69	2.06	26.63	34.93
CAA in Leiden	46.50	2.22	42.21	50.89	46.46	2.23	42.21	50.75
Estimated - infection prevalence								
Kato-Katz in Burundi	-26.35	3.11	-32.72	-20.69	-34.02	3.04	-39.97	-27.96
CCA in Burundi	-11.99	3.51	-18.90	-5.31	12.80	3.56	5.63	19.44
CCA in Leiden	-4.75	3.52	-11.90	1.91	-10.09	3.46	-16.92	-3.40
CAA in Leiden	13.36	3.66	6.06	20.41	5.68	3.49	-1.38	12.40

*Abbreviations: SD* standard deviation, *LBCI* lower Bayesian 95% credible interval, *UBCI* upper Bayesian 95% credible interval

### Proportion of trace results truly positive

**Fig. 4:**
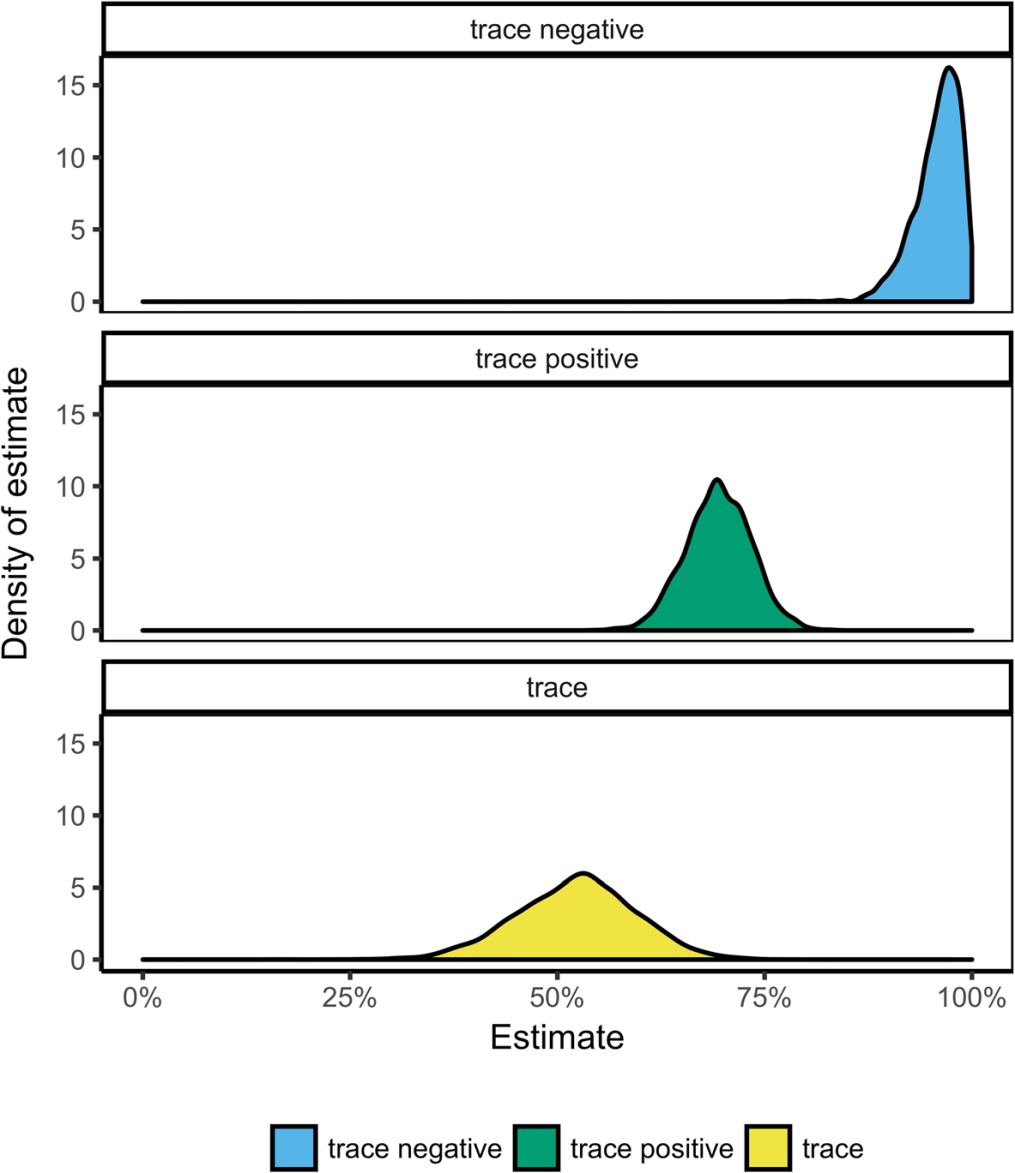
Distribution of positive predictive values (PPV) of CCA performed in Burundi from a Bayesian LCA of 398 pupils in 8 schools when trace was considered negative (top), positive (middle) and for traces only (bottom)

Our model suggests that 95.8% (BCI: 89.4–99.6%) of the positive CCAB results were truly infection positive when CCA trace was considered as negative (Table 4, Fig. 4). However, when CCA trace was considered as positive, 69.4% (BCI: 61.7–77.1%) of the positive CCA results were estimated to be truly positive. Combining these results suggested that 52.2% (BCI: 37.8–65.8%) of the CCAB ‘trace’ results were indeed true positive results, although the BCIs on this estimate were relatively wide.

### Estimating test properties when specificity of CAA fixed at 100%

We repeated the analyses above after fixing the specificity of CAA to 100%, to estimate the other tests’ properties if all the CAA positive results were indeed true infections. The sensitivity estimates for all tests were lower when CAA specificity was set at 100%, than in the original analyses where CAA specificity was allowed to vary. However, examination of the 95% BCI’s of the differences between models indicated that only the changes in CCAB and CCAL sensitivities were significant. Specificity estimates were comparable between analyses, and all 95% BCI’s of the differences between models straddled zero (see Additional file 1: Table S6 for parameter estimates and Additional file 1: Table S7 for BCI’s of differences between parameter estimates).

The estimated infection prevalence from these models was substantially higher than when specificity of CAA was allowed to vary, but strikingly similar whether trace was considered as negative or positive, at 53.4% (BCI: 48.5–58.3%; Fig. 3; Additional file 1: Tables S6 and S7) when CCA trace was considered negative and 52.3% (BCI: 47.4–57.2%) when CCA trace was considered positive. In the model with trace considered negative, all tests significantly underestimated infection prevalence, but in the model with trace as positive, CCAB and CAA gave prevalence estimates not significantly different from estimated infection prevalence (Additional file 1: Table S8).

## Discussion

With data from 398 children, we used LCA to assess the performance of three different assays for *S. mansoni* infection, with one assay being independently performed in two different locations. We confirmed the long-agreed low sensitivity of microscopic KK egg counts for low intensity infections without multiple days of Kato-Katz, and then quantified the higher test sensitivity of both CCA and CAA antigen tests. We compared estimated infection prevalence with that estimated using each test’s results and demonstrated that CCA performed much better in detecting infection than KK. CAA showed very high sensitivity. Although it is currently strictly a laboratory-based assay, it could be a promising confirmatory test in the future.

We analysed the data using LCA in a Bayesian framework. The use of LCA avoids having to make assumptions as to which test, or test combinations, perform as a ‘gold standard’; that is, the best measure of prevalence (as was done in two recent reviews [35, 36]). Rather, the LCA combined all the available data to generate an estimate of infection prevalence. Performing the analyses in a Bayesian framework had two main advantages. First, we could use a strong prior on the parameter we had most confidence in, which was the specificity of KK microscopy, as the visual detection of an egg indicates actual infection unless laboratory/administrative error or contamination has occurred. Indeed, the estimates of KK specificity were above the mode of the Bayesian prior values, suggesting that the data supported our assumption, and this is further supported by the model being robust to the choice of prior. Secondly, the added flexibility from the Bayesian output enabled us to easily compare estimated prevalence by the different assays to estimated infection prevalence by LCA. Because national control programs will typically measure prevalence rather than each individual’s infection status, this approach allowed us to assess test performance for way it will most commonly be used in the field.

The sensitivity of KK was much lower than that of any other test. Although this result was expected in an area of low mean infection intensity [7], the magnitude by which KK underestimated infection prevalence remained striking, and the performance of the KK test is clearly sub-optimal for programmes such as that of Burundi. KK remains a valuable tool in areas of higher infection intensity [7], where prevalence estimates are more consistent with those based on CCA testing. Additionally, KK remains a useful tool for concurrent detection of soil-transmitted helminths. However, our data and analyses indicate that it should be used in concert with the CCA assay if low *S. mansoni* intensity is expected.

CCA specificity assuming traces are negative was comparable to that of KK, but its estimated sensitivity was much greater. Treating CCA trace as positive in Burundi further increased the sensitivity of CCA, but at the cost of reduced specificity. The PPV estimates we have calculated suggested that a substantial proportion of the CCA trace results, around half but perhaps 35–65%, were indeed true infections. Consequently, prevalence estimated using CCAB with trace as negative likely underestimated infection prevalence, while CCAB with trace as positive possibly overestimated infection prevalence. However, if the CAA-positive results were indeed all true positives, then CCAB with trace as positive may have accurately reflected infection prevalence in the eight schools.

There is currently no field test to accurately determine schistosomiasis prevalence in the population, i.e. there is no ‘gold standard’ field diagnosis, but it is evident that the CCA test performs better than KK in diagnosing *S. mansoni* infection in low prevalence/low intensity settings. Additionally, the relative ease of use of CCA compared to KK means that CCA may be more suitable for use in the community, perhaps in test and treat strategies [4], especially given that the cost of individual CCA tests is decreasing over time.

The sensitivity estimates of CAA were very high, above 90%, although the specificity of CAA was relatively low, at 75% when trace was considered negative and 85% when trace was considered positive. The high sensitivity of CAA was consistent with the raw data, where CAA detected the highest proportion of infections positive by KK. However, the relatively low specificity estimate for CAA was driven by children for whom CAA was the only positive test. Thus, this low specificity could be a result of false positives by CAA, with all other tests being true negatives, or could result from CAA being true positive while all other tests are false negatives. This is an expected output from an LCA when only one test indicates infection, unless a strong prior is indicated for that test. Data from other studies [13, 16, 37] have indicated very high specificity of CAA. Therefore, we re-ran the analyses with CCA specificity fixed at 100%, which resulted in lower sensitivity estimates for all tests, and much lower estimates for CCAB and CCAL. These results indicated that the prevalence estimates from CCAB with trace as positive may indeed reflect infection prevalence in the population.

The difference between CCA results in Burundi and Leiden is striking, in particular the fewer number of trace readings in Leiden. Low levels of within and between observer variation in reading of CCA cassettes has been observed in field evaluation [8]. However, this study considered observers who were in the same location and presumably trained together. Although teams were provided with pictures of standard references, the differences observed may have been due to between-group differences in CCA interpretation, especially around the trace results. That 98% of the trace results in Burundi were assessed as within one scale point of trace in Leiden, and that 84% of the non-trace results were the same between Burundi and Leiden, suggests that the differences may have been due to interpretation of trace results. Efforts are ongoing to reduce variability in readings by changing the cassette or developing a mobile optical scanner or software to read cassettes. Additionally, we cannot discount the possibility that transportation affected the urine samples, even though CCA is an extremely stable carbohydrate and all storage and transportation protocols were followed. Nevertheless, CCA measured in both Burundi and Leiden performed substantially better at estimating prevalence than the current alternative field diagnostic, KK. It remains to be seen if high numbers of trace readings will be a common feature of CCA tests in low-intensity settings but it is clear that CCA outperforms Kato-Katz in these difficult to assess areas. We recommend that CCA trace results be explicitly recorded in the field to enable comparison of prevalence estimates considering trace as negative and as positive.

Although there is clearly a need for very large-scale studies using many diagnostics, this study is a valuable contribution to the literature, providing more knowledge on the performance of CCA in low intensity settings. The main strength of this study is the inclusion of the CAA results to provide more information on each individual’s infection status and to improve the robustness of the model. Another important aspect of the study is that it took place in a low-intensity setting; many other CCA studies have taken place in higher intensity settings. In Burundi, only 16% of schools had at least one pupil testing positive by KK. The cost of CAA laboratory testing in Leiden meant that samples from only eight schools could be sent for CAA evaluation. Consequently, there was a substantial chance that random sampling of schools would lead to very little meaningful data being available for the KK tests. Hence, the decision was taken to purposively select the eight schools, resulting in a prevalence by KK of 6.8% compared to 1.5% across all 170 schools that were assessed by both CCA and KK. Although purposive sampling may induce some biases in the data, the prevalence by KK in the selected schools was still low, giving us confidence that the results presented here can be generalised to low prevalence and intensity settings. The sample size of 398 pupils is also perhaps why attempts to fit both trace negative and trace positive results into a single model failed, as did attempts to fit multiple covariances into the model a model with CCA, and we instead fitted only the covariance term that resulted in the lowest DIC. Although larger sample sizes were not possible because of the cost of processing CAA, the inclusion of this extra test adds insight and robustness that would not be possible from simply comparing the larger number of CCA and KK results obtained in the field, and which are reported in detail elsewhere [26].

It is clear from these results that there is much more residual *S. mansoni* infection in Burundi than KK indicates [27], with the models suggesting infection prevalence of 33% when trace was negative and 41% when trace was positive. The higher prevalence with trace as positive is expected as there was more agreement between tests when trace was considered positive. However, these trace-positive infections are likely to be of low intensity, and, although there may always be morbidity associated with any *Schistosoma* infection [38], the need to treat in areas where most positive children are trace-positive for morbidity prevention may be less acute than in areas with higher intensities of infection. Even considering CCA trace as negative would give prevalence estimates three to four times higher than found by KK.

## Conclusions

The appropriate tool to test for *S. mansoni* infection is dependent on the stage of advancement of the control program [39]. Both KK and CCA perform well when average infection intensities are high, which is typical at the beginning of morbidity control programs. Because of its greater sensitivity, CCA is clearly more suitable for established control programmes, as in Burundi, where KK is likely to perform poorly, and as countries decide to move towards *Schistosoma* elimination. CAA is another available diagnostic tool, but it is currently expensive and solely laboratory-based. In many diseases, it is common to use a step-wise combination of diagnostics to confirm diagnosis, and this is also likely to be true in low-intensity schistosomiasis. It seems clear that CCA, recently recommended by the WHO, is a useful component of the diagnostic toolbox, particularly for mapping and screening programmes, but it should not be seen as the only tool. As schistosomiasis elimination programmes become more mature the number of tools will hopefully increase, and CAA may also prove to be a valuable field tool in the future. We would caution against comparing new diagnostics or mapping tools only against an imperfect standard test (either KK in the case of *S. mansoni* or urine filtration in the case of *S. haematobium*) when use of other means, such as LCA, will yield more appropriate and reliable information about relative diagnostic performance of better tests.

## Additional file

Additional file 1: **Table S1.** Bayesian Deviance Information Criterion (DIC) from models with different covariances fitted. **Table S2.** Summary statistics by school and prevalence estimates for each separate test by school. **Table S3**. Comparison of CCA results in Burundi and Leiden. **Table S4.** Test result combinations overall and by school, when CCA trace was considered negative and positive. **Table S5**. Estimate and 95% BCIs of difference between same estimates from trace negative and trace positive models presented in Table 4. **Table S6.** Output from LCA when specificity of CAA fixed to 100%. **Table S7.** Estimate and 95% BCIs of difference between same estimates from different models. **Table S8** Estimated test and infection prevalence when specificity of CAA fixed to 100%. **Fig. S1.** Sensitivity of models to prior assumptions. **Code S1.** Code for running the LCA in R2OpenBugs (DOCX 845 kb)
