# Supplementary material for: Latent class analysis to evaluate performance of point-of-care CCA for low-intensity Schistosoma mansoni infections in Burundi
Source: Parasit Vectors. 2018 Feb 23;11:111. doi: 10.1186/s13071-018-2700-4 (PMC5824563; doi:10.1186/s13071-018-2700-4)
Supplement: Supplementary file 1 — Table S1. Bayesian Deviance Information Criterion (DIC) from models with different covariances fitted. Table S2. Summary statistics by school and prevalence estimates for each separate test by school. Table S3. Comparison of CCA results in Burundi and Leiden. Table S4. Test result combinations overall and by school, when CCA trace was considered negative and positive. Table S5. Estimate and 95% BCIs of difference between same estimates from trace negative and trace positive models presented in Table 4. Table S6. Output from LCA when specificity of CAA fixed to 100%. Table S7. Estimate and 95% BCIs of difference between same estimates from different models. Table S8 Estimated test and infection prevalence when specificity of CAA fixed to 100%. Fig. S1. Sensitivity of models to prior assumptions. Code S1. Code for running the LCA in R2OpenBugs (DOCX 845 kb) [file 13071_2018_2700_MOESM1_ESM.docx]

**Additional file 1: Table S1.** Bayesian Deviance Information Criterion (DIC) from models with different covariances fitted. * indicates the final model (the model with the smallest DIC).

| Covariance | DIC Trace neg | DIC Trace pos |
| --- | --- | --- |
| No covariances | 308.3 | 321.1 |
| KK & CCAB | 307.4 | 323.8 |
| KK & CCAL | 310.9 | 323.9 |
| KK & CAA | 310.1 | 320.3 |
| CCAB & CCAL | 310.3 | 319.2* |
| CCAB & CAA | 310.8 | 323.6 |
| CCAL & CAA | 302.3* | 321.8 |
|  |  |  |
| KK & CCAB + CCAL & CAA | 304.6 | 325.2 |
| KK & CAA + CCAB & CCAL | 312.0 | 321.0 |

**Additional file 1: Table S2.** Summary statistics by school and prevalence estimates for each separate test by school. KK: Kato-Katz, CCABtn: CCA in Burundi with trace as negative; CCABtp: CCA in Burundi with trace as positive, CCALtn: CCA in Leiden with trace as negative, CCALtp: CCA in Leiden with trace as positive, CAA: CAA in Leiden.

|  |  |  |  |  | Prevalence | |  |  |  |
| --- | --- | --- | --- | --- | --- | --- | --- | --- | --- |
| School Code | Number pupil | Percent girl | mean age | KK | CCABtn | CCABtp | CCALtn | CCALtp | CAA |
| 48 | 50 | 50.0% | 12.5 | 2.0% | 44.0% | 90.0% | 60.0% | 64.0% | 78.0% |
| 60 | 50 | 48.0% | 13.1 | 2.0% | 16.0% | 40.0% | 28.0% | 28.0% | 48.0% |
| 82 | 49 | 49.0% | 13.3 | 4.1% | 26.5% | 69.4% | 22.4% | 24.5% | 61.2% |
| 83 | 50 | 50.0% | 13.2 | 6.0% | 30.0% | 44.0% | 30.0% | 30.0% | 36.0% |
| 102 | 50 | 50.0% | 13.0 | 0.0% | 0.0% | 12.0% | 0.0% | 0.0% | 6.0% |
| 163 | 50 | 50.0% | 13.3 | 4.0% | 4.0% | 38.0% | 8.0% | 12.0% | 18.0% |
| 168 | 50 | 54.0% | 13.2 | 20.0% | 38.0% | 70.0% | 52.0% | 56.0% | 74.0% |
| 343 | 49 | 59.2% | 13.7 | 16.3% | 10.2% | 65.3% | 26.5% | 30.6% | 51.0% |

**Additional file 1: Table S3.** Comparison of CCA results in Burundi and Leiden. Cells showing the same results in Burundi and Leiden are coloured in green.

|  | | CCA result in Leiden (CCAL) | | | | | |
| --- | --- | --- | --- | --- | --- | --- | --- |
|  |  | **0** | **trace** | **1** | **2** | **3** | **Total** |
| CCA result in Burundi (CCAB) | **0** | 176 | 0 | 7 | 1 | 1 | **185** |
|  | **trace** | 88 | 8 | 31 | 2 | 0 | **129** |
|  | **1** | 7 | 1 | 30 | 3 | 0 | **41** |
|  | **2** | 4 | 0 | 9 | 10 | 1 | **24** |
|  | **3** | 1 | 0 | 2 | 8 | 8 | **19** |
|  | **Total** | **276** | **9** | **79** | **24** | **10** | **398** |

**Additional file 1: Table S4** Test result combinations overall and by school, when CCA trace was considered negative (top) and positive (bottom). The 0/1 for each test denotes whether that test result was negative or positive, with 0 indicating negative and 1 indicating positive. KK: Kato-Katz, CCAB: CCA performed in Burundi, CCAL: CCA performed in Leiden, CAA: CAA in Leiden. The data split by school was inputted in the Bayesian Latent Class Analysis.

| **CCA trace negative test results** | | | | SchoolCode | | | | | | | | Total |
| --- | --- | --- | --- | --- | --- | --- | --- | --- | --- | --- | --- | --- |
| **KK** | **CCAB** | **CCAL** | **CAA** | 1 | 2 | 3 | 4 | 5 | 6 | 7 | 8 |  |
| 1 | 1 | 1 | 1 | 0 | 1 | 1 | 3 | 0 | 1 | 7 | 2 | 15 |
| 1 | 1 | 1 | 0 | 0 | 0 | 0 | 0 | 0 | 0 | 0 | 0 | 0 |
| 1 | 1 | 0 | 1 | 0 | 0 | 0 | 0 | 0 | 0 | 0 | 0 | 0 |
| 1 | 1 | 0 | 0 | 0 | 0 | 1 | 0 | 0 | 0 | 0 | 0 | 1 |
| 1 | 0 | 1 | 1 | 0 | 0 | 0 | 0 | 0 | 0 | 2 | 4 | 6 |
| 1 | 0 | 1 | 0 | 0 | 0 | 0 | 0 | 0 | 0 | 0 | 0 | 0 |
| 1 | 0 | 0 | 1 | 1 | 0 | 0 | 0 | 0 | 1 | 0 | 1 | 3 |
| 1 | 0 | 0 | 0 | 0 | 0 | 0 | 0 | 0 | 0 | 1 | 1 | 2 |
| 0 | 1 | 1 | 1 | 18 | 6 | 4 | 10 | 0 | 0 | 11 | 3 | 52 |
| 0 | 1 | 1 | 0 | 1 | 1 | 0 | 1 | 0 | 0 | 1 | 0 | 4 |
| 0 | 1 | 0 | 1 | 3 | 0 | 5 | 0 | 0 | 0 | 0 | 0 | 8 |
| 0 | 1 | 0 | 0 | 0 | 0 | 2 | 1 | 0 | 1 | 0 | 0 | 4 |
| 0 | 0 | 1 | 1 | 9 | 4 | 6 | 1 | 0 | 1 | 5 | 4 | 30 |
| 0 | 0 | 1 | 0 | 2 | 2 | 0 | 0 | 0 | 2 | 0 | 0 | 6 |
| 0 | 0 | 0 | 1 | 8 | 13 | 14 | 4 | 3 | 6 | 12 | 11 | 71 |
| 0 | 0 | 0 | 0 | 8 | 23 | 16 | 30 | 47 | 38 | 11 | 23 | 196 |
| Total |  |  |  | 50 | 50 | 49 | 50 | 50 | 50 | 50 | 49 | 398 |
|  |  |  |  |  |  |  |  |  |  |  |  |  |
| **CCA trace positive test results** | | | | SchoolCode | | | | | | | | Total |
| **KK** | **CCAB** | **CCAL** | **CAA** | 1 | 2 | 3 | 4 | 5 | 6 | 7 | 8 |  |
| 1 | 1 | 1 | 1 | 0 | 1 | 1 | 3 | 0 | 1 | 8 | 6 | 20 |
| 1 | 1 | 1 | 0 | 0 | 0 | 0 | 0 | 0 | 0 | 0 | 0 | 0 |
| 1 | 1 | 0 | 1 | 1 | 0 | 0 | 0 | 0 | 0 | 0 | 0 | 1 |
| 1 | 1 | 0 | 0 | 0 | 0 | 1 | 0 | 0 | 0 | 0 | 0 | 1 |
| 1 | 0 | 1 | 1 | 0 | 0 | 0 | 0 | 0 | 0 | 1 | 0 | 1 |
| 1 | 0 | 1 | 0 | 0 | 0 | 0 | 0 | 0 | 0 | 0 | 0 | 0 |
| 1 | 0 | 0 | 1 | 0 | 0 | 0 | 0 | 0 | 1 | 0 | 1 | 2 |
| 1 | 0 | 0 | 0 | 0 | 0 | 0 | 0 | 0 | 0 | 1 | 1 | 2 |
| 0 | 1 | 1 | 1 | 28 | 10 | 9 | 10 | 0 | 1 | 17 | 9 | 84 |
| 0 | 1 | 1 | 0 | 4 | 1 | 0 | 1 | 0 | 2 | 1 | 0 | 9 |
| 0 | 1 | 0 | 1 | 9 | 3 | 11 | 2 | 0 | 4 | 7 | 6 | 42 |
| 0 | 1 | 0 | 0 | 3 | 5 | 12 | 6 | 6 | 11 | 2 | 11 | 56 |
| 0 | 0 | 1 | 1 | 0 | 0 | 2 | 1 | 0 | 1 | 1 | 0 | 5 |
| 0 | 0 | 1 | 0 | 0 | 2 | 0 | 0 | 0 | 1 | 0 | 0 | 3 |
| 0 | 0 | 0 | 1 | 1 | 10 | 7 | 2 | 3 | 1 | 3 | 3 | 30 |
| 0 | 0 | 0 | 0 | 4 | 18 | 6 | 25 | 41 | 27 | 9 | 12 | 142 |
| Total |  |  |  | 50 | 50 | 49 | 50 | 50 | 50 | 50 | 49 | 398 |

**Additional file 1: Table S5**. Estimate and 95% BCIs of difference between same estimates from trace negative and trace positive models presented in table 4.

|  | Estimate of difference  Final model: trace neg - trace pos |
| --- | --- |
| Sensitivity: KK | 0.9% (-8.1%, 10.2%) |
| Sensitivity: CCAB | -30.4% (-42.8%, -18.1%) |
| Sensitivity: CCAL | 7.5% (-7.0%, 21.7%) |
| Sensitivity: CAA | -1.5% (-9.6%, 6.4%) |
|  |  |
| Specificity: KK | -0.4% (-3.5%, 2.8%) |
| Specificity: CCAB | 26.4% (20.0%, 33.4%) |
| Specificity: CCAL | 0.5% (-2.8%, 3.9%) |
| Specificity: CAA | -10.7% (-19.4%, -1.9%) |
|  |  |
| Prevalence | -7.6% (-15.3%, 0.3%) |

**Additional file 1: Table S6.** output from LCA when specificity of CAA fixed to 100%

|  | CCA trace negative | CCA trace positive |
| --- | --- | --- |
| Sensitivity (95% BCI) |  |  |
| Kato-Katz in Burundi | 10.5% (7.2%, 14.4%) | 12.5% (8.2%, 17.2%) |
| CCA in Burundi | 37.4% (30.9%, 44.3%) | 80.2% (74.1%, 85.7%) |
| CCA in Leiden | 49.8% (42.4%, 57.4%) | 56.9% (50.0%, 64.0%) |
| CAA in Leiden | 84.6% (77.6%, 90.6%) | 84.4% (77.5%, 90.6%) |
|  |  |  |
| covariance CCAL & CAA | 1.6% (0.8%, 2.3%) |  |
| covariance CCAB & CCAL |  | 0.1% (0.0%, 0.4%) |
|  |  |  |
| Specificity (95% BCI) |  |  |
| Kato-Katz in Burundi | 99.0% (97.3%, 99.9%) | 98.5% (95.8%, 99.8%) |
| CCA in Burundi | 99.0% (96.6%, 99.9%) | 74.5% (67.7%, 81.2%) |
| CCA in Leiden | 98.2% (95.4%, 99.9%) | 95.7% (92.7%, 98.1%) |
| CAA in Leiden | 100.0% (100.0%, 100.0%) | 100.0% (100.0%, 100.0%) |
|  |  |  |
| covariance CCAL & CAA | 0.1% (0.0%, 0.4%) |  |
| covariance CCAB & CCAL |  | 2.4% (1.0%, 4.2%) |
|  |  |  |
| PPV of CCA in Burundi (95% BCI) |  |  |
| Trace as negative | 97.7% (92.8%, 99.9%) | |
| Trace as positive | 77.5% (71.0%, 83.4%) | |
| Trace results only | 65.0% (53.5%, 75.1%) | |

**Additional file 1: Table S7.** Estimate and 95% BCIs of difference between same estimates from different models. Left: difference between trace negative and trace positive models where specificity of CAA fixed to 100%. Middle: difference between trace negative final model (table 4) and trace negative model with CAA specificity fixed to 100% (table S6). Right: difference between trace positive final model (table 4) and trace positive model with CAA specificity fixed to 100% (table S6).

|  | 100% spec models:  trace neg - trace pos | Trace neg:  final model - 100% spec model | Trace pos:  final model - 100% spec model |
| --- | --- | --- | --- |
| Sensitivity: KK | -1.9% (-7.8%, 3.7%) | 5.4% (-2.3%, 13.6%) | 2.5% (-4.6%, 10.2%) |
| Sensitivity: CCAB | -42.8% (-51.5%, -33.5%) | 23.7% (10.6%, 36.6%) | 11.3% (3.3%, 18.7%) |
| Sensitivity: CCAL | -7.1% (-17.4%, 2.7%) | 29.7% (16.0%, 42.0%) | 15.1% (3.3%, 26.3%) |
| Sensitivity: CAA | 0.2% (-9.0%, 9.2%) | 5.7% (-2.8%, 14.1%) | 7.4% (-1.8%, 16.3%) |
| covariance - Sensitivity: CCAL & CAA |  | -0.9% (-2.0%, 0.2%) |  |
| covariance - Sensitivity: CCAB & CCAL |  |  | 0.1% (-0.3%, 0.6%) |
|  |  |  |  |
| Specificity: KK | 0.6% (-1.7%, 3.5%) | -1.9% (-4.8%, 0.6%) | -1.0% (-3.7%, 2.3%) |
| Specificity: CCAB | 24.5% (17.8%, 31.5%) | -0.3% (-2.8%, 2.3%) | -2.2% (-11.5%, 7.1%) |
| Specificity: CCAL | 2.5% (-1.2%, 6.1%) | -0.9% (-4.1%, 2.6%) | 1.1% (-2.6%, 4.8%) |
| Specificity: CAA | N/A | -25.4% (-31.7%, -18.8%) | -14.7% (-20.7%, -9.0%) |
| covariance - Specificity: CCAL & CAA |  | 0.3% (-0.2%, 1.0%) |  |
| covariance - Specificity: CCAB & CCAL |  |  | -2.1% (-3.9%, -0.5%) |
|  |  |  |  |
| Prevalence | 1.1% (-5.8%, 7.8%) | -20.2% (-27.5%, -12.8%) | -11.5% (-19.1%, -4.1%) |

**Additional file 1: Table S8.** Estimated test and infection prevalence when specificity of CAA fixed to 100%

|  | trace negative | | | | |  | | trace positive | | | | |  |
| --- | --- | --- | --- | --- | --- | --- | --- | --- | --- | --- | --- | --- | --- |
|  | | mean | sd | LBCI | UBCI | |  | | mean | sd | LBCI | UBCI | |
| Infection prevalence | | 53.37% | 2.48% | 48.51% | 58.25% | |  | | 52.25% | 2.48% | 47.43% | 57.15% | |
|  | |  |  |  |  | |  | |  |  |  |  | |
| Estimated test prevalence | |  |  |  |  | |  | |  |  |  |  | |
| Kato-Katz in Burundi | | 6.83% | 1.23% | 4.52% | 9.30% | |  | | 6.78% | 1.24% | 4.52% | 9.30% | |
| CCA in Burundi | | 21.13% | 1.93% | 17.59% | 25.13% | |  | | 53.52% | 2.21% | 49.00% | 58.04% | |
| CCA in Leiden | | 28.40% | 2.05% | 24.37% | 32.41% | |  | | 30.66% | 2.07% | 26.63% | 34.93% | |
| CAA in Leiden | | 46.41% | 2.24% | 41.96% | 50.75% | |  | | 46.50% | 2.25% | 42.08% | 51.01% | |
|  | |  |  |  |  | |  | |  |  |  |  | |
| Estimated - infection prevalence | | |  |  |  | |  | |  |  |  |  | |
| Kato-Katz in Burundi | | -46.55% | 2.76% | -51.78% | -41.02% | | | | -45.47% | 2.78% | -51.16% | -39.95% | |
| CCA in Burundi | | -32.24% | 3.12% | -38.38% | -26.16% | | | | 1.28% | 3.33% | -5.55% | 7.75% | |
| CCA in Leiden | | -24.97% | 3.24% | -31.44% | -18.53% | | | | -21.59% | 3.22% | -27.93% | -15.27% | |
| CAA in Leiden | | -6.96% | 3.31% | -13.27% | -0.38% | |  | | -5.75% | 3.31% | -12.23% | 0.79% | |

**Additional file 1: Figure S1.** Sensitivity of models to prior assumptions. The models were not very sensitive to the choice of priors, when trace was both negative (top) and positive (bottom). Each box shows a single parameter estimate with associated 95% BCI for multiple outputs from models with different priors. The top line in each box shows the parameter estimate from the model presented in the manuscript with informative priors, and the bottom line in each box shows the same parameter estimate when uninformative Beta(1, 1) priors were used for all parameters; lines in-between show parameter estimates when the prior of a single parameter was set to Beta(1, 1). Prevalence estimates in each school are shown as these were outputted from the model and overall prevalence was calculated from these posterior distributions. KK: Kato-Katz performed in Burundi, CCAB – CCA performed in Burundi, CCAL – CCA performed in Leiden, CAA – CAA performed in Leiden.


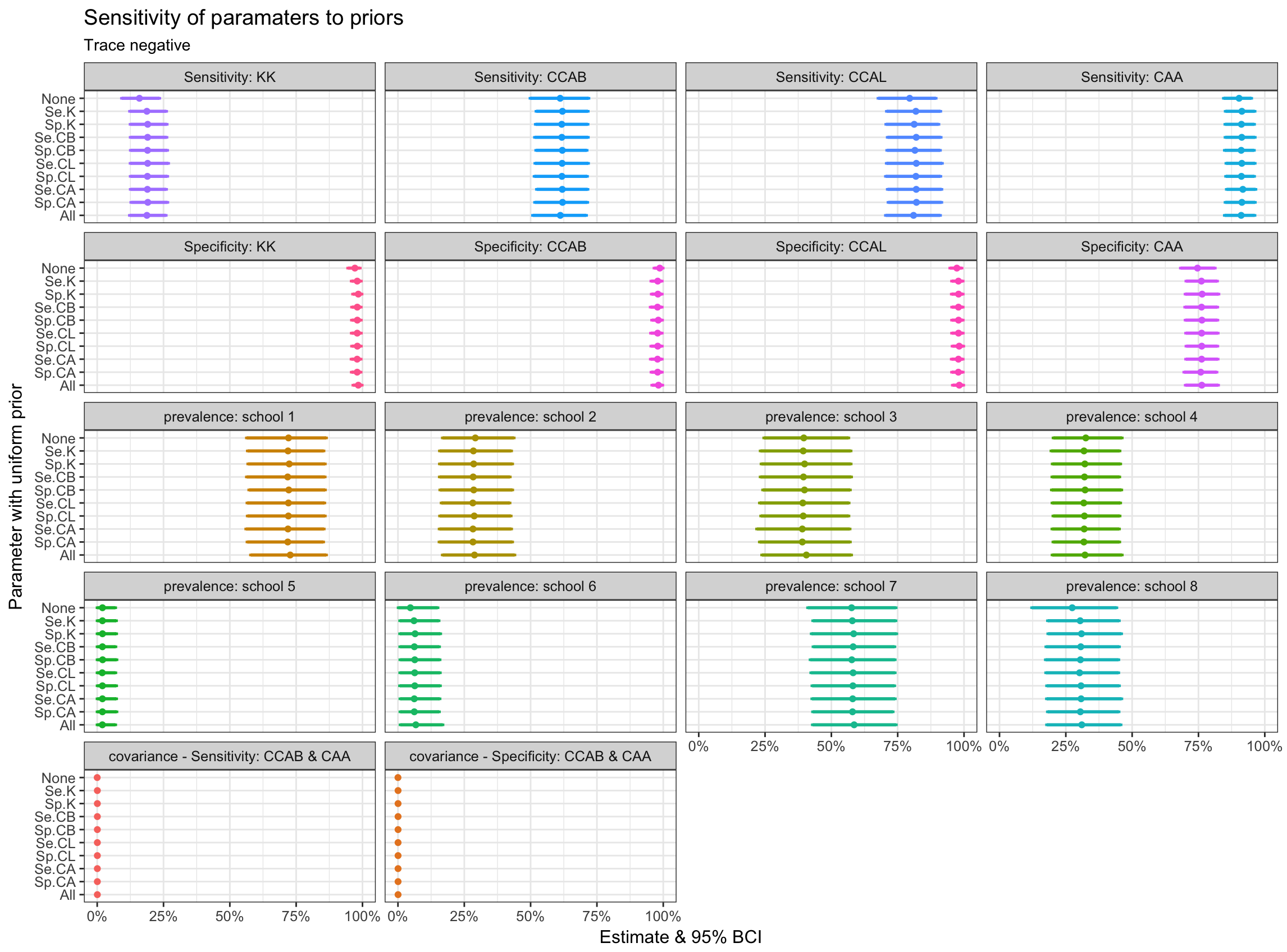

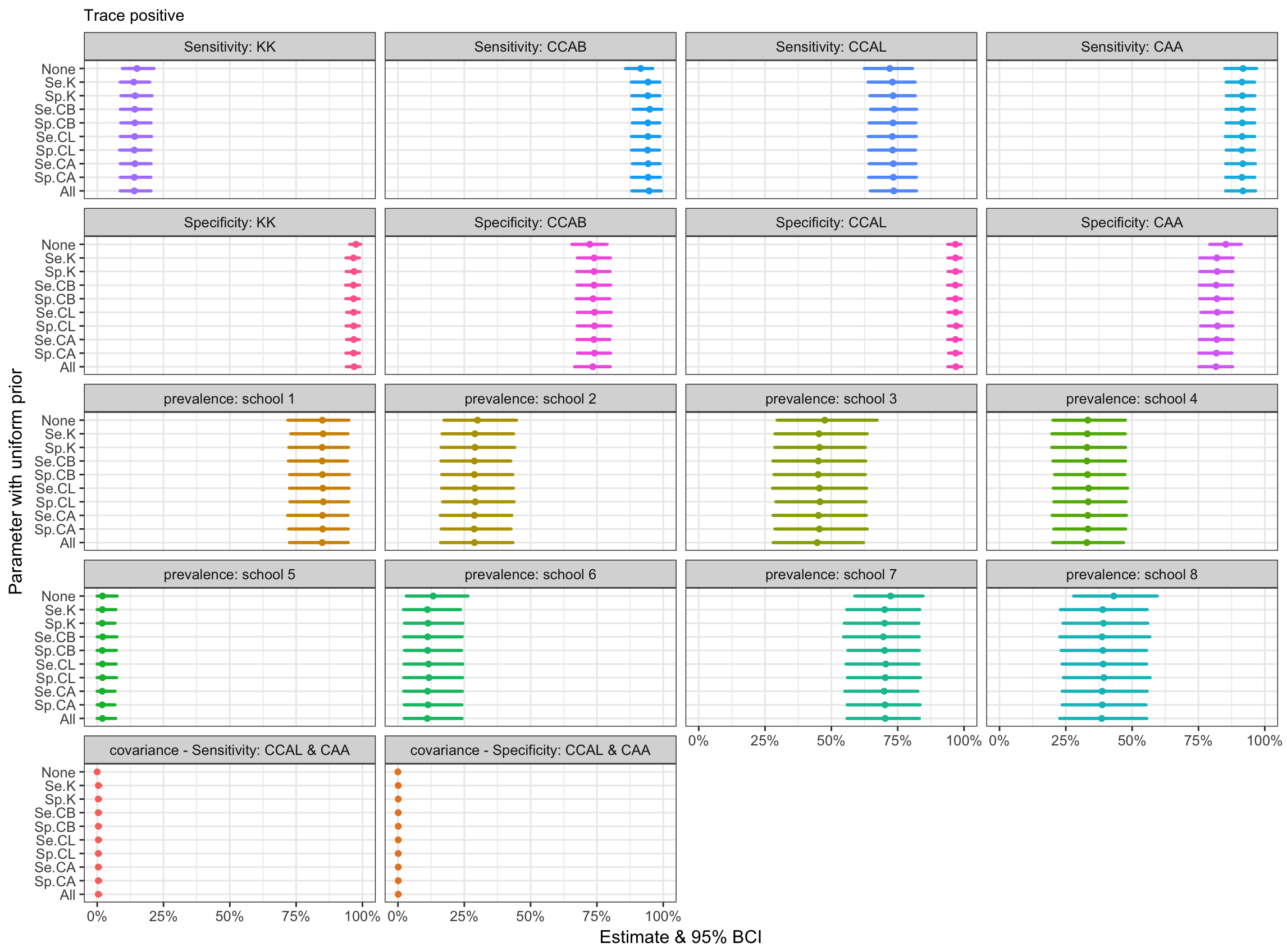


**Additional file 1: Code S1.** Code for running the LCA in R2OpenBugs

LCA.model <- function(){

#====likelihood==========================

for (i in 1:8){

y[i, 1] <- prev[i] * (Se.K * Se.CB * Se.CL * Se.CA + c.Se.K.CB + c.Se.K.CL + c.Se.K.CA + c.Se.CB.CL + c.Se.CB.CA + c.Se.CL.CA) + (1 - prev[i]) * ((1 - Sp.K) * (1 - Sp.CB) * (1 - Sp.CL) * (1 - Sp.CA) + c.Sp.K.CB + c.Sp.K.CL + c.Sp.K.CA + c.Sp.CB.CL + c.Sp.CB.CA + c.Sp.CL.CA)

y[i, 2] <- prev[i] * (Se.K * Se.CB * Se.CL * (1 - Se.CA) + c.Se.K.CB + c.Se.K.CL -c.Se.K.CA + c.Se.CB.CL -c.Se.CB.CA -c.Se.CL.CA) + (1 - prev[i]) * ((1 - Sp.K) * (1 - Sp.CB) * (1 - Sp.CL) * Sp.CA + c.Sp.K.CB + c.Sp.K.CL -c.Sp.K.CA + c.Sp.CB.CL -c.Sp.CB.CA -c.Sp.CL.CA)

y[i, 3] <- prev[i] * (Se.K * Se.CB * (1 - Se.CL) * Se.CA + c.Se.K.CB -c.Se.K.CL + c.Se.K.CA -c.Se.CB.CL + c.Se.CB.CA -c.Se.CL.CA) + (1 - prev[i]) * ((1 - Sp.K) * (1 - Sp.CB) * Sp.CL * (1 - Sp.CA) + c.Sp.K.CB -c.Sp.K.CL + c.Sp.K.CA -c.Sp.CB.CL + c.Sp.CB.CA -c.Sp.CL.CA)

y[i, 4] <- prev[i] * (Se.K * Se.CB * (1 - Se.CL) * (1 - Se.CA) + c.Se.K.CB -c.Se.K.CL -c.Se.K.CA -c.Se.CB.CL -c.Se.CB.CA + c.Se.CL.CA) + (1 - prev[i]) * ((1 - Sp.K) * (1 - Sp.CB) * Sp.CL * Sp.CA + c.Sp.K.CB -c.Sp.K.CL -c.Sp.K.CA -c.Sp.CB.CL -c.Sp.CB.CA + c.Sp.CL.CA)

y[i, 5] <- prev[i] * (Se.K * (1 - Se.CB) * Se.CL * Se.CA -c.Se.K.CB + c.Se.K.CL + c.Se.K.CA -c.Se.CB.CL -c.Se.CB.CA + c.Se.CL.CA) + (1 - prev[i]) * ((1 - Sp.K) * Sp.CB * (1 - Sp.CL) * (1 - Sp.CA) -c.Sp.K.CB + c.Sp.K.CL + c.Sp.K.CA -c.Sp.CB.CL -c.Sp.CB.CA + c.Sp.CL.CA)

y[i, 6] <- prev[i] * (Se.K * (1 - Se.CB) * Se.CL * (1 - Se.CA) -c.Se.K.CB + c.Se.K.CL -c.Se.K.CA -c.Se.CB.CL + c.Se.CB.CA -c.Se.CL.CA) + (1 - prev[i]) * ((1 - Sp.K) * Sp.CB * (1 - Sp.CL) * Sp.CA -c.Sp.K.CB + c.Sp.K.CL -c.Sp.K.CA -c.Sp.CB.CL + c.Sp.CB.CA -c.Sp.CL.CA)

y[i, 7] <- prev[i] * (Se.K * (1 - Se.CB) * (1 - Se.CL) * Se.CA -c.Se.K.CB -c.Se.K.CL + c.Se.K.CA + c.Se.CB.CL -c.Se.CB.CA -c.Se.CL.CA) + (1 - prev[i]) * ((1 - Sp.K) * Sp.CB * Sp.CL * (1 - Sp.CA) -c.Sp.K.CB -c.Sp.K.CL + c.Sp.K.CA + c.Sp.CB.CL -c.Sp.CB.CA -c.Sp.CL.CA)

y[i, 8] <- prev[i] * (Se.K * (1 - Se.CB) * (1 - Se.CL) * (1 - Se.CA) -c.Se.K.CB -c.Se.K.CL -c.Se.K.CA + c.Se.CB.CL + c.Se.CB.CA + c.Se.CL.CA) + (1 - prev[i]) * ((1 - Sp.K) * Sp.CB * Sp.CL * Sp.CA -c.Sp.K.CB -c.Sp.K.CL -c.Sp.K.CA + c.Sp.CB.CL + c.Sp.CB.CA + c.Sp.CL.CA)

y[i, 9] <- prev[i] * ((1 - Se.K) * Se.CB * Se.CL * Se.CA -c.Se.K.CB -c.Se.K.CL -c.Se.K.CA + c.Se.CB.CL + c.Se.CB.CA + c.Se.CL.CA) + (1 - prev[i]) * (Sp.K * (1 - Sp.CB) * (1 - Sp.CL) * (1 - Sp.CA) -c.Sp.K.CB -c.Sp.K.CL -c.Sp.K.CA + c.Sp.CB.CL + c.Sp.CB.CA + c.Sp.CL.CA)

y[i, 10] <- prev[i] * ((1 - Se.K) * Se.CB * Se.CL * (1 - Se.CA) -c.Se.K.CB -c.Se.K.CL + c.Se.K.CA + c.Se.CB.CL -c.Se.CB.CA -c.Se.CL.CA) + (1 - prev[i]) * (Sp.K * (1 - Sp.CB) * (1 - Sp.CL) * Sp.CA -c.Sp.K.CB -c.Sp.K.CL + c.Sp.K.CA + c.Sp.CB.CL -c.Sp.CB.CA -c.Sp.CL.CA)

y[i, 11] <- prev[i] * ((1 - Se.K) * Se.CB * (1 - Se.CL) * Se.CA -c.Se.K.CB + c.Se.K.CL -c.Se.K.CA -c.Se.CB.CL + c.Se.CB.CA -c.Se.CL.CA) + (1 - prev[i]) * (Sp.K * (1 - Sp.CB) * Sp.CL * (1 - Sp.CA) -c.Sp.K.CB + c.Sp.K.CL -c.Sp.K.CA -c.Sp.CB.CL + c.Sp.CB.CA -c.Sp.CL.CA)

y[i, 12] <- prev[i] * ((1 - Se.K) * Se.CB * (1 - Se.CL) * (1 - Se.CA) -c.Se.K.CB + c.Se.K.CL + c.Se.K.CA -c.Se.CB.CL -c.Se.CB.CA + c.Se.CL.CA) + (1 - prev[i]) * (Sp.K * (1 - Sp.CB) * Sp.CL * Sp.CA -c.Sp.K.CB + c.Sp.K.CL + c.Sp.K.CA -c.Sp.CB.CL -c.Sp.CB.CA + c.Sp.CL.CA)

y[i, 13] <- prev[i] * ((1 - Se.K) * (1 - Se.CB) * Se.CL * Se.CA + c.Se.K.CB -c.Se.K.CL -c.Se.K.CA -c.Se.CB.CL -c.Se.CB.CA + c.Se.CL.CA) + (1 - prev[i]) * (Sp.K * Sp.CB * (1 - Sp.CL) * (1 - Sp.CA) + c.Sp.K.CB -c.Sp.K.CL -c.Sp.K.CA -c.Sp.CB.CL -c.Sp.CB.CA + c.Sp.CL.CA)

y[i, 14] <- prev[i] * ((1 - Se.K) * (1 - Se.CB) * Se.CL * (1 - Se.CA) + c.Se.K.CB -c.Se.K.CL + c.Se.K.CA -c.Se.CB.CL + c.Se.CB.CA -c.Se.CL.CA) + (1 - prev[i]) * (Sp.K * Sp.CB * (1 - Sp.CL) * Sp.CA + c.Sp.K.CB -c.Sp.K.CL + c.Sp.K.CA -c.Sp.CB.CL + c.Sp.CB.CA -c.Sp.CL.CA)

y[i, 15] <- prev[i] * ((1 - Se.K) * (1 - Se.CB) * (1 - Se.CL) * Se.CA + c.Se.K.CB + c.Se.K.CL -c.Se.K.CA + c.Se.CB.CL -c.Se.CB.CA -c.Se.CL.CA) + (1 - prev[i]) * (Sp.K * Sp.CB * Sp.CL * (1 - Sp.CA) + c.Sp.K.CB + c.Sp.K.CL -c.Sp.K.CA + c.Sp.CB.CL -c.Sp.CB.CA -c.Sp.CL.CA)

y[i, 16] <- prev[i] * ((1 - Se.K) * (1 - Se.CB) * (1 - Se.CL) * (1 - Se.CA) + c.Se.K.CB + c.Se.K.CL + c.Se.K.CA + c.Se.CB.CL + c.Se.CB.CA + c.Se.CL.CA) + (1 - prev[i]) * (Sp.K * Sp.CB * Sp.CL * Sp.CA + c.Sp.K.CB + c.Sp.K.CL + c.Sp.K.CA + c.Sp.CB.CL + c.Sp.CB.CA + c.Sp.CL.CA)

# tying y to the data

result[i, 1 : 16] ~ dmulti(y[i, 1 : 16], tested[i]) # observed data

}

#===prior distributions ===============================================

for (i in 1 : 8) {prev[i] ~ dbeta(1, 1) }

Se.K ~ dbeta(1.43, 1.29) # p1 # 95% sure > 0.1. Mode at 0.6.

Sp.K ~ dbeta(21.20, 2.06) # p6 # 95% sure > 0.8. Mode at 0.95.

Se.CB ~ dbeta(3.05, 1.51) # p2 # 95% sure > 0.3. Mode at 0.8..

Sp.CB ~ dbeta(5.38, 1.49) # p4 # 95% sure > 0.5. Mode at 0.9.

Se.CL ~ dbeta(3.05, 1.51) # p2 # 95% sure > 0.3. Mode at 0.8.

Sp.CL ~ dbeta(5.38, 1.49) # p4 # 95% sure > 0.5. Mode at 0.9.

Se.CA ~ dbeta(3.05, 1.51) # p2 # 95% sure > 0.3. Mode at 0.8.

Sp.CA ~ dbeta(5.38, 1.49) # p4 # 95% sure > 0.5. Mode at 0.9.

c.Se.K.CB ~ dunif(0, U.c.Se.K.CB)

c.Se.K.CL ~ dunif(0, U.c.Se.K.CL)

c.Se.K.CA ~ dunif(0, U.c.Se.K.CA)

c.Se.CB.CL ~ dunif(0, U.c.Se.CB.CL)

c.Se.CB.CA ~ dunif(0, U.c.Se.CB.CA)

c.Se.CL.CA ~ dunif(0, U.c.Se.CL.CA)

c.Sp.K.CB ~ dunif(0, U.c.Sp.K.CB)

c.Sp.K.CL ~ dunif(0, U.c.Sp.K.CL)

c.Sp.K.CA ~ dunif(0, U.c.Sp.K.CA)

c.Sp.CB.CL ~ dunif(0, U.c.Sp.CB.CL)

c.Sp.CB.CA ~ dunif(0, U.c.Sp.CB.CA)

c.Sp.CL.CA ~ dunif(0, U.c.Sp.CL.CA)

# limits for the priors - setting the relevant param to zero removes the covariance from the model

U.c.Se.K.CB <- min(Se.K, min(Se.CB, min(Se.CL, Se.CA))) - Se.K * Se.CB * Se.CL * Se.CA - c.Se.K.CL - c.Se.K.CA - c.Se.CB.CL - c.Se.CB.CA - c.Se.CL.CA

# U.c.Se.K.CB <- 0

U.c.Se.K.CL <- min(Se.K, min(Se.CB, min(Se.CL, Se.CA))) - Se.K * Se.CB * Se.CL * Se.CA - c.Se.K.CB - c.Se.K.CA - c.Se.CB.CL - c.Se.CB.CA - c.Se.CL.CA

# U.c.Se.K.CL <- 0

U.c.Se.K.CA <- min(Se.K, min(Se.CB, min(Se.CL, Se.CA))) - Se.K * Se.CB * Se.CL * Se.CA - c.Se.K.CB - c.Se.K.CL - c.Se.CB.CL - c.Se.CB.CA - c.Se.CL.CA

# U.c.Se.K.CA <- 0

U.c.Se.CB.CL <- min(Se.K, min(Se.CB, min(Se.CL, Se.CA))) - Se.K * Se.CB * Se.CL * Se.CA - c.Se.K.CB - c.Se.K.CL - c.Se.K.CA - c.Se.CB.CA - c.Se.CL.CA

# U.c.Se.CB.CL <- 0

U.c.Se.CB.CA <- min(Se.K, min(Se.CB, min(Se.CL, Se.CA))) - Se.K * Se.CB * Se.CL * Se.CA - c.Se.K.CB - c.Se.K.CL - c.Se.K.CA - c.Se.CB.CL - c.Se.CL.CA

# U.c.Se.CB.CA <- 0

U.c.Se.CL.CA <- min(Se.K, min(Se.CB, min(Se.CL, Se.CA))) - Se.K * Se.CB * Se.CL * Se.CA - c.Se.K.CB - c.Se.K.CL - c.Se.K.CA - c.Se.CB.CL - c.Se.CB.CA

# U.c.Se.CL.CA <- 0

U.c.Sp.K.CB <- min(Sp.K, min(Sp.CB, min(Sp.CL, Sp.CA))) - Sp.K * Sp.CB * Sp.CL * Sp.CA - c.Sp.K.CL - c.Sp.K.CA - c.Sp.CB.CL - c.Sp.CB.CA - c.Sp.CL.CA

# U.c.Sp.K.CB <- 0

U.c.Sp.K.CL <- min(Sp.K, min(Sp.CB, min(Sp.CL, Sp.CA))) - Sp.K * Sp.CB * Sp.CL * Sp.CA - c.Sp.K.CB - c.Sp.K.CA - c.Sp.CB.CL - c.Sp.CB.CA - c.Sp.CL.CA

# U.c.Sp.K.CL <- 0

U.c.Sp.K.CA <- min(Sp.K, min(Sp.CB, min(Sp.CL, Sp.CA))) - Sp.K * Sp.CB * Sp.CL * Sp.CA - c.Sp.K.CB - c.Sp.K.CL - c.Sp.CB.CL - c.Sp.CB.CA - c.Sp.CL.CA

# U.c.Sp.K.CA <- 0

U.c.Sp.CB.CL <- min(Sp.K, min(Sp.CB, min(Sp.CL, Sp.CA))) - Sp.K * Sp.CB * Sp.CL * Sp.CA - c.Sp.K.CB - c.Sp.K.CL - c.Sp.K.CA - c.Sp.CB.CA - c.Sp.CL.CA

# U.c.Sp.CB.CL <- 0

U.c.Sp.CB.CA <- min(Sp.K, min(Sp.CB, min(Sp.CL, Sp.CA))) - Sp.K * Sp.CB * Sp.CL * Sp.CA - c.Sp.K.CB - c.Sp.K.CL - c.Sp.K.CA - c.Sp.CB.CL - c.Sp.CL.CA

# U.c.Sp.CB.CA <- 0

U.c.Sp.CL.CA <- min(Sp.K, min(Sp.CB, min(Sp.CL, Sp.CA))) - Sp.K * Sp.CB * Sp.CL * Sp.CA - c.Sp.K.CB - c.Sp.K.CL - c.Sp.K.CA - c.Sp.CB.CL - c.Sp.CB.CA

# U.c.Sp.CL.CA <- 0

}

LCA.inits <- function(){list(prev = runif(8, 0, 1),

Se.K = runif(1, 0.1, 0.5),

Sp.K = runif(1, 0.9, 1),

Se.CB = runif(1, 0.5, 0.9),

Sp.CB = runif(1, 0.6, 1),

Se.CL = runif(1, 0.5, 0.9),

Sp.CL = runif(1, 0.6, 1),

Se.CA = runif(1, 0.5, 0.9),

Sp.CA = runif(1, 0.6, 1),

c.Se.K.CB = 0,

c.Se.K.CL = 0,

c.Se.K.CA = 0,

c.Se.CB.CL = 0,

c.Se.CB.CA = 0,

c.Se.CL.CA = 0,

c.Sp.K.CB = 0,

c.Sp.K.CL = 0,

c.Sp.K.CA = 0,

c.Sp.CB.CL = 0,

c.Sp.CB.CA = 0,

c.Sp.CL.CA = 0

)}

# parameters to monitor

LCA.monitor <- c("prev",

"Se.K", "Sp.K",

"Se.CB", "Sp.CB",

"Se.CL", "Sp.CL",

"Se.CA", "Sp.CA",

"c.Se.K.CB", "c.Se.K.CL",

"c.Se.K.CA", "c.Se.CB.CL",

"c.Se.CB.CA", "c.Se.CL.CA",

"c.Sp.K.CB", "c.Sp.K.CL",

"c.Sp.K.CA", "c.Sp.CB.CL",

"c.Sp.CB.CA", "c.Sp.CL.CA"

)

LCA.out <- bugs(LCA.data, LCA.inits, LCA.monitor, LCA.model,

n.iter=1000, n.burnin=100, n.thin=25, n.chains=3, debug = T)
